# Profilin as a regulator of the membrane-actin cytoskeleton interface in plant cells

**DOI:** 10.3389/fpls.2013.00512

**Published:** 2013-12-19

**Authors:** Tiantian Sun, Shanwei Li, Haiyun Ren

**Affiliations:** Key Laboratory of Cell Proliferation and Regulation Biology of Ministry of Education, College of Life Science, Beijing Normal UniversityBeijing, China

**Keywords:** actin cytoskeleton, profilin, plasma membrane, organelle, vesicle, plants

## Abstract

Membrane structures and cytoskeleton dynamics are intimately inter-connected in the eukaryotic cell. Recently, the molecular mechanisms operating at this interface have been progressively addressed. Many experiments have revealed that the actin cytoskeleton can interact with membranes through various discrete membrane domains. The actin-binding protein, profilin has been proven to inhibit actin polymerization and to promote F-actin elongation. This is dependent on many factors, such as the profilin/G-actin ratio and the ionic environment of the cell. Additionally, profilin has specific domains that interact with phosphoinositides and poly-L-proline rich proteins; theoretically, this gives profilin the opportunity to interact with membranes, and a large number of experiments have confirmed this possibility. In this article, we summarize recent findings in plant cells, and discuss the evidence of the connections among actin cytoskeleton, profilin and biomembranes through direct or indirect relationships.

## INTRODUCTION

The membrane is a lipid bilayer that functions to divide and separate the cells and organelles. It undergoes many dynamic morphological changes during cellular processes such as endocytosis, exocytosis, vesicular transport, and morphogenesis. Growing evidence has demonstrated that actin cytoskeleton dynamics are involved in these processes. However, the interactions between microfilaments and membranes vary in different cell types and locations. Some cytoskeletal elements may interact with membranes directly. Transmembrane proteins can regulate membrane-cytoskeleton interactions directly or indirectly through adaptor proteins or adaptor complexes. Furthermore, some proteins have domains that can associate with the membrane, and domains that can interact with cytoskeletal components. These are the main types of membrane-cytoskeleton interactions (Doherty and McMahon, 2008). The extracellular matrix (ECM) of animals mainly consists of proteinaceous materials. However, the plant cell wall, which deviates plant cell from spherical shapes, mainly consists of carbohydrates. This implies that there are differences in the intermolecular interactions that occur in membrane-cytoskeleton of animal and plant. In mammals, cytoskeletal proteins that can function as adaptors, such as talin (Heise et al., 1991), vinculin (Geiger et al., 1980), and filamin (Stossel et al., 2001) bind the actin cytoskeleton to membranes; homologs of these proteins are absent from plants (Hussey et al., 2002). There are many plant-specific linker molecules. For example, myosin VIII binds directly or indirectly to plasma membrane-localized callose synthase complexes (Verma and Hong, 2001; Ostergaard et al., 2002) and it also binds to actin filaments in the cytoplasm, which implies that myosin VIII associates plasma membrane with actin filaments in plants. Moreover, a plant-specific Networked (NET) superfamily of actin-binding proteins is found in *Arabidopsis*. Members of the NET superfamily localize to the actin cytoskeleton and specify different membrane compartments. NET1A is located at the plasma membrane and binds directly to actin filaments through a novel actin-binding domain. The NET superfamily is grouped into four phylogenetic clades, and other members have functions at the tonoplast, nuclear membrane, and pollen tube plasma membrane, which suggest that this superfamily is involved in regulating actin-membrane interactions (Deeks et al., 2012).

A large amount of literature has fostered our current understanding of the membrane, the actin cytoskeleton, and of actin-binding proteins that mediate membrane and actin cytoskeleton components. Profilins are actin-binding proteins, and have the capacity to interact with three classes of ligands. In addition to G-actin, they also associate with poly-L-proline (PLP) which can interact with the binding cleft formed from the N-terminal and C-terminal helices of profilin (Metzler et al., 1994; Mahoney et al., 1999) and phosphoinositides (Gibbon and Staiger, 2000; Jockusch et al., 2007) which offers the possibility that profilin interacts with the membrane. In recent years, much evidence has been verified that profilins can interact with membranes directly or indirectly. In this review, we will summarize recent findings and focus predominantly on the functions of profilins in the direct or indirect relationships among actin cytoskeleton, profilin and membranes in plant cells.

## MULTIFUNCTIONAL PROFILINS

**FIGURE 1:**
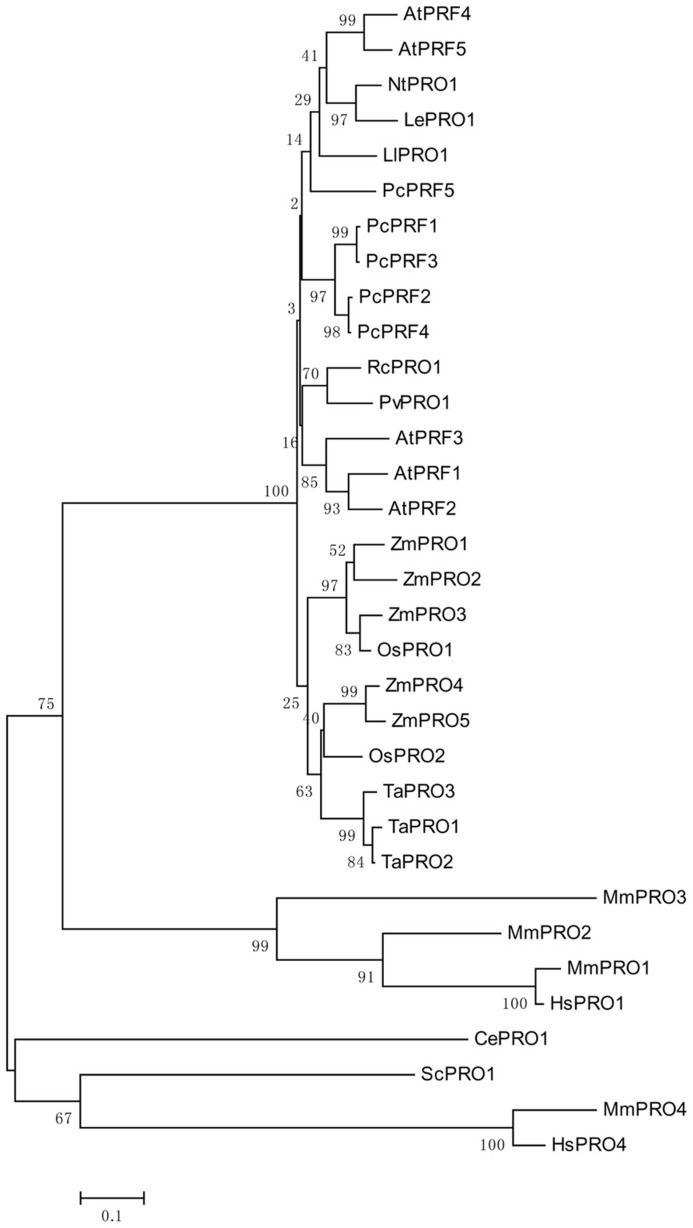
**An unrooted phylogenetic tree of profilins.** The plant genes are *Arabidopsis thaliana* AtPRF1–AtPRF5 (AT2G19760, AT4G29350, AT5G56600, AT4G29340, AT2G19770), *Petroselinum crispum* PcPRF1–PcPRF5 (AY900012-AY900016), *Zea mays* ZmPRO1–ZmPRO5 (X73279, X73280, X73281, AF032370, AF201459), *Oryza sativa* OsPRO1–OsPRO2 (LOC_Os10g17680, LOC_Os06g05880), *Triticum aestivum* TaPRO1–TaPRO3 (X89825-X89827), *Nicotiana tabacum* NtPRO1 (pronp1 AJ130969), tomato LePRO1 (U50195), *Ricinus communis* RcPRO1 (AF092547), *Phaseolus vulgaris* PvPRO1 (CAA57508), *Lilium longiflorum* LlPRO1 (AF200184). Selected fungal and metazoan sequences are included: *Mus musculus* MmPRO1–MmPRO4 (NP_035202, NP_062283, NP_083579, AK013595), *Homo sapiens* HsPRO1 and HsPRO4 (BC057828, BC029523), *Caenorhabditis elegans* CePRO1 (PFN-1, NP_493258) *Saccharomyces cerevisiae* ScPRO1 (PFY1, NP_014765). The percentage of replicate trees in which the associated taxa clustered together in the bootstrap test (1000 replicates) is shown next to the branches (Felsenstein, 1985). The tree is drawn to scale, with branch lengths in the same units as those of the evolutionary distances used to infer the phylogenetic tree.

Genomic DNA sequences of putative profilins contain three exons; these may be separated by introns of different sizes (Huang et al., 1996), and are dispersed throughout the genome. Comparing the amino acid sequences of different profilins reveal that profilins have less than 25% identity across different kingdoms (Pollard and Quirk, 1994), but are highly conserved, with at least 70% identity, across various plant species (Mittermann et al., 1995; Vidali et al., 1995). This is consistent with the analysis of the phylogenetic tree shown in **Figure 1**. Although the secondary and tertiary structures of all profilins are well conserved (Fedorov et al., 1997; Thorn et al., 1997; Jockusch et al., 2007), the fact that many varieties of profilins isoforms exist in different species, and even in the same organism, may indicate that members of the profilin family have diverse functions. Plant profilins are from multigene families and can be divided into two major groups: the vegetative group, in which profilins exist extensively and are constitutively expressed in all plant tissues; and the reproductive group, where profilins are expressed in reproductive tissues (Kandasamy et al., 2002). The *Arabidopsis* profilin family includes five highly different isoforms: AtPRF1–AtPRF5; AtPRF1–AtPRF3 belong to the vegetative class, and AtPRF4 and AtPRF5 to the reproductive class (Christensen et al., 1996; Kandasamy et al., 2002). AtPRF1 has much higher affinities for both PLP and G-actin than AtPRF2 (Wang et al., 2009). The tobacco profilin gene, pronp1, is prominently expressed in mature pollen, elongating pollen tubes, and the root hairs of developing seedlings. Pronp1 represents a unique profilin as it has activities in two kinds of tip-growing cells, the pollen tubes and root hairs, which rapidly regulate the organization of the actin cytoskeleton (Swoboda et al., 2001). In tomato, LePRO1 was found to be expressed only in pollen grains, and not in other parts of the anther or in other organs using a non-radioactive labeling method (Yu et al., 1998). RcPRO1, a *Ricinus communis* phloem profilin, is expressed in epidermal, cortex, pith, and xylem tissue. In the sieve-tube exudates, RcPRO1 has 15-fold molar excess to actin, which suggests that actin filament formation is blocked in the assimilate stream (Schobert et al., 2000). In maize, five profilins have been identified (ZmPRO1–ZmPRO5); ZmPRO1–ZmPRO3 are major profilin isoforms of a pollen-abundant class, whereas ZmPRO4 and ZmPRO5 appear to be members of a predominantly endosperm profilin class. Furthermore, ZmPRO1 inhibits hydrolysis of membrane phosphatidylinositol-4, 5-bisphosphate (PIP_2_) by phospholipase C more effectively than ZmPRO5. Conversely, ZmPRO5 has higher affinity for PLP and sequesters more monomeric actin to inhibit actin polymerization better than ZmPRO1 (Staiger et al., 1993; Kovar et al., 2000). Currently, there are over 400 profilins from 100 plant species, which are effective at NCBI GenBank database (Pruitt et al., 2007; Jimenez-Lopez et al., 2012). All of the above evidences support that profilins are multifunctional proteins according to their expressions and locations.

## PROFILIN IS INVOLVED IN PLASMA MEMBRANE-ACTIN CYTOSKELETON INTERACTIONS

Binding interactions between the plasma membrane and the actin cytoskeleton define cell functions such as cytoplasmic streaming, cytokinesis, and endocytosis. Profilin is one of the crucial linkers of the membrane-cytoskeleton interaction. The inherent interaction of the actin cytoskeleton with the plasma membrane is through the relationship between actin-binding proteins and PIP_2_, which itself localizes to the inner side of the plasma membrane (Nebl et al., 2000; Caroni, 2001). PIP_2_ can bind to transmembrane adhesion protein, and also interacts with several actin-binding proteins including profilin (Goldschmidt-Clermont et al., 1990; Heiska et al., 1998; Couchman et al., 2002). Immunofluorescence analysis revealed that at the plasma membrane of maize root cells, PIP_2_ is targeted to discrete domains that resemble profilin-enriched domains. PIP_2_redistributes and the actin cytoskeleton remodels following treatment with phospholipase C activator mastoparan (Baluska et al., 2001). Therefore, profilin may be a linker between the plasma membrane and actin cytoskeleton through PIP_2_. Furthermore, profilins can interact with the proteins that contain PLP stretches of at least eight to ten prolines in continuous or discontinuous sequences (Schluter et al., 1997). In eukaryotes, formins are a group of actin-binding proteins that contain the FH1 domain with different numbers of PLP stretches; they are considered to act as morphological regulation proteins that direct the assembly of unbranched actin filaments (Paul and Pollard, 2009). Profilins or actin/profilin complexes can interact with the PLP stretches of different formins to promote actin filament polymerization (Chang et al., 1997; Pruyne et al., 2002; Kovar et al., 2006; Paul and Pollard, 2009). Additionally, type I formins contain an N-terminal transmembrane domains; this is the region of formin association with the plasma membrane in plants (Cvrckova et al., 2004). For example, in *Arabidopsis*, formin homology 6 (AtFH6) interacting with profilin locates at the plasma membrane and is uniformly distributed (Favery et al., 2004). Furthermore, AtFH1 and AtFH5 are reported to associate with the cell membrane (Banno and Chua, 2000; Cheung and Wu, 2004; Ingouff et al., 2005). This verifies that plant type I formins are likely to be membrane-bound, with AtFH8 being the exception, as it is targeted to the nuclear envelope (Xue et al., 2011). The site of the profilin binding FH1 PLP tracks is on the opposite face of the actin binding site of profilin (Schutt et al., 1993), and this explains why profilin can bind PLP and actin simultaneously without mutual influence (Tanaka and Shibata, 1985; Perelroizen et al., 1994). Profilin has an indirect connection and possibly acts as a regulator in the linkage of the plasma membrane and the actin cytoskeleton.

The plant cell is able to defend itself from infection by exogenous fungi. During this process, the cytoskeleton reorganizes and the papilla localizes at penetration sites, this leads to a thick cell wall being formed to prevent pathogen ingress (Schmelzer, 2002). Material is site-directed to arrive at positions around the fungal infection structure beneath the cell wall, and the actin filament and microtubule re-orientate their structures toward the penetration site (Schmelzer, 2002; Takemoto et al., 2003). In cultured parsley cells, undergoing attack from infection with the oomycetous plant pathogen *Phytophthora infestans*, profilin is expressed and accumulates at the site of infection on the plasma membrane, and the actin cables focus at the penetration site where Rop GTPases also accumulate (Schutz et al., 2006). In addition, in developing microspores and mature pollen of *Zea mays*, profilin is associated with the plasma membrane (von Witsch et al., 1998). Profilin accumulates in the tip zone near the plasma membrane in root hairs of *Arabidopsis* (Braun et al., 1999; Baluska et al., 2000). These results suggest that profilins play a role in both signal transduction and linkage between the plasma membrane and actin cytoskeleton

## PROFILIN IS INVOLVED IN ORGANELLE LOCATION WITH THE ACTIN CYTOSKELETON

There is much evidence, that in various eukaryotic cells the cytoskeleton is involved in organelle movements. In plant cells, the role of the actin cytoskeleton in organelle movements has been reported for movements of chloroplasts (Kandasamy and Meagher, 1999), the endoplasmic reticulum (ER; Boevink et al., 1998), and the Golgi apparatus (Boevink et al., 1998; Nebenfuhr et al., 1999).

In *Arabidopsis*, CHUP1 (*Chloroplast unusual positioning 1*) which is a 112 kDa protein that is closely related with chloroplast movement (Kasahara et al., 2002; Oikawa et al., 2003) is directly targeted to the outer envelope of the chloroplast; this is dependent on its N-terminus domain (Oikawa et al., 2003). In addition to the N-terminus domain, the CHUP1 protein has four other domains, including two leucine-zippers, an actinin-type actin binding domain (Gimona et al., 2002), and a proline-rich motif (PRM) that is similar to PRM1 identified as a profilin binding motif (Holt and Koffer, 2001). A fusion protein which includes GST and the actin binding domain of CHUP1 can bind F-actin *in vitro* (Oikawa et al., 2003). The *in vitro* biochemical analyzes revealed that CHUP1 interacts with profilin as a modulator of actin polymerization through the PRM of C-terminal part of CHUP1 (CHUP1-CT). The experiment of CHUP1-CT titrated to a mixture of profilin and actin confirmed that the trimeric complex of actin, profilin, and CHUP1-CT is more stable than the individual binary complex. Though CHUP1 can bind F-actin directly, profilin has been reported to enhance the connection between chloroplasts and actin filaments (Schmidt von Braun and Schleiff, 2008).

Although profilin can bind to formin, the type II formins do not contain the transmembrane domains present in type I formins (Cvrckova et al., 2004). In rice, like other plant type II formins, formin homology 5 (FH5) has a characteristic N-terminal phosphatase tensin (PTEN)-related domain that may interact with membranes (Cvrckova et al., 2004). The experiments of transiently expressing the PTEN-RFP fusion protein in tobacco (*Nicotiana tabacum*) cells and immunostaining analysis using rice leaf cells revealed that the PTEN-like domain of FH5 is sufficient to confer localization of the protein to the chloroplast surface. This suggests that the PTEN domain of FH5 may be a bridge between chloroplasts and the actin cytoskeleton (Zhang et al., 2011). Furthermore, FH5 was capable of nucleating actin assembly from the actin/profilin complex *in vitro* biochemical analyzes (Yang et al., 2011; Zhang et al., 2011). Therefore, profilin is indirectly involved in the localization of chloroplast to the actin filaments. In *Arabidopsis*, observations of living cells in stable transgenic plants revealed that 35S:: GFP-AtPRF1 forms a filamentous network likely associated with actin filaments; this was verified by treatment with latrunculin A, and through a recovery experiment involving the removal of latrunculin A. Whereas, 35S:: GFP-AtPRF2 forms polygonal meshes resembling ER in the same latrunculin A treatment conditions (Wang et al., 2009). Furthermore, in plants, profilins possibly participate in the linkage of the nuclear envelope and the actin cytoskeleton during the interphase of *Arabidopsis*; this is because AtFH8 locates primarily to the nuclear envelope at this stage (Xue et al., 2011).

## PROFILIN IS INVOLVED IN VESICLE TRAFFICKING

Profilins are known to play an important role in endocytosis and membrane trafficking in lower eukaryotes (Wolven et al., 2000; Pearson et al., 2003). In mammalian cells, profilins may also be involved in membrane trafficking. It has been reported that profilin 1 exists in budding Golgi vesicles, and that dynamin 2 recruitment to the Golgi is dependent on profilin 1 (Dong et al., 2000). Moreover, in mammalian cells, there are multiple phosphoinositide 3-kinases (PI3Ks), and these can be grouped into three main classes. Class I and II PI3Ks can induce receptor-dependent trafficking processes, such as phagocytosis. Class III PI3Ks, which represent the most ancient form of PI3Ks, and are the only ones conserved in lower eukaryotes, mammals, and plants. Class III PI3Ks mainly regulate receptor-independent trafficking events, such as endocytic membrane traffic (Lindmo and Stenmark, 2006). In animal cells, PI3Ks have been reported to play many different roles in vesicle trafficking, and inhibition of PI3Ks induces the inhibition of clathrin-dependent endocytosis (Martys et al., 1996; Spiro et al., 1996). In plant cells, Class III PI3K protein complexes may have a regulatory function during vesicle trafficking (Matsuoka et al., 1995; Kim et al., 2001; Jung et al., 2002). In *Phaseolus vulgaris*, in addition to the N- and C-terminal PLP-binding domain, profilin has a domain around Tyr72; this can recognize and bind PLP and PI3K (Aparicio-Fabre et al., 2006). Profilin can bind directly to Class III PI3Ks in a manner reliant upon the tyrosine phosphorylation status of the PLP domain in profilin. This interaction between profilin and Class III PI3Ks suggests that profilin may participate in membrane trafficking, and may act as a linker between the endocytic pathway and the actin reorganization dynamics (Aparicio-Fabre et al., 2006).

With advances in biotechnology, diverse pharmaceutical drugs have been used to study the interaction between vesicular trafficking and cytoskeleton. Brefeldin A (BFA) is a drug that inhibits the recycling of vesicular trafficking, and disrupts secretion in yeast, mammalian, and plant cells (Vogel et al., 1993; Samaj et al., 2004; Citterio et al., 2008; Robinson et al., 2008). In *Arabidopsis* roots, BFA-compartments can be formed due to the accumulation of trans-Golgi network (TGN) secretory and recycling vesicles, which gather together following BFA treatment (Geldner et al., 2003). During this process, profilin 2 is up-regulated and accumulates in the BFA-compartments, which then interacts with the actin to remodel the actin cytoskeleton. This study suggested that profilin 2 may bridge vesicular trafficking to the actin cytoskeleton in a BFA-dependent manner (Takac et al., 2011). **Table 1** lists the profilins cited in the present article and emphasizes some of their cellular functions. Therefore, the recently investigated interactions between membranes and the actin cytoskeleton have revealed profilins to be of particular interest, this is because they may act as linkers and regulate communication and cooperation between the two cellular members in plants. Currently available studies suggest that diverse interaction mechanisms are required to satisfy the different structural and dynamic requirements of particular systems. Future research is required to unravel how membrane-actin cytoskeleton interactions are regulated through profilins and their different ligands.

**Table 1:** Profilin and its cellular functions in plant cells.

Profilin involving in the cellular pathway	Profilins	Cells or ligands	Reference
Plasma membrane-actin cytoskeleton interaction	ZmPRO3	Root cells of maize PIP2	Baluska et al. (2001)
	AtPRF1 etc	*Arabidopsis* seed endosperm, root cells etc AtFH1, AtFH5, and AtFH6	Banno and Chua (2000); Cheung and Wu (2004), Ingouff et al. (2005), Favery et al. (2004)
	PcPRF1	Cultured parsley cells	Schutz et al. (2006)
Organelles location with the actin cytoskeleton	Profilins from *Arabidopsis*	*Arabidopsis* mesophyll protoplasts CHUP1	Oikawa et al. (2003); Schmidt von Braun and Schleiff (2008)
	NA	Rice leaf cells OsFH5	Zhang et al. (2011); Yang et al. (2011)
	AtPRF2	*Arabidopsis* epidermal cells, trichomes, stem epidermal cells ER**	Wang et al. (2009)
	NA	*Arabidopsis* root cells AtFH8	Xue et al. (2011)
Vesicle trafficking with the actin cytoskeleton	PvPRO1	*Phaseolus vulgaris* root nodules Class III PI3Ks	Aparicio-Fabre et al. (2006)
	AtPRF2	*Arabidopsis* roots TGN	Takac et al. (2011)

*NA, not available.*

## Conflict of Interest Statement

The authors declare that the research was conducted in the absence of any commercial or financial relationships that could be construed as a potential conflict of interest.
